# A Biomimetic Control Method Increases the Adaptability of a Humanoid Robot Acting in a Dynamic Environment

**DOI:** 10.3389/fnbot.2019.00070

**Published:** 2019-08-28

**Authors:** Marie Claire Capolei, Emmanouil Angelidis, Egidio Falotico, Henrik Hautop Lund, Silvia Tolu

**Affiliations:** ^1^Automation and Control Group, Department of Electrical Engineering, Technical University of Denmark, Copenhagen, Denmark; ^2^Landesforschungsinstitut des Freistaats Bayern, An-Institut, Technical University of Munich, Munich, Germany; ^3^The BioRobotics Institute, Scuola Superiore Sant'Anna, Pisa, Italy

**Keywords:** biomimetic, cerebellar control, motor learning, humanoid robot, adaptive system, forward model, bio-inspired, neurorobotics

## Abstract

One of the big challenges in robotics is to endow agents with autonomous and adaptive capabilities. With this purpose, we embedded a cerebellum-based control system into a humanoid robot that becomes capable of handling dynamical external and internal complexity. The cerebellum is the area of the brain that coordinates and predicts the body movements throughout the body-environment interactions. Different biologically plausible cerebellar models are available in literature and have been employed for motor learning and control of simplified objects. We built the canonical cerebellar microcircuit by combining machine learning and computational neuroscience techniques. The control system is composed of the adaptive cerebellar module and a classic control method; their combination allows a fast adaptive learning and robust control of the robotic movements when external disturbances appear. The control structure is built offline, but the dynamic parameters are learned during an online-phase training. The aforementioned adaptive control system has been tested in the Neuro-robotics Platform with the virtual humanoid robot iCub. In the experiment, the robot iCub has to balance with the hand a table with a ball running on it. In contrast with previous attempts of solving this task, the proposed neural controller resulted able to quickly adapt when the internal and external conditions change. Our bio-inspired and flexible control architecture can be applied to different robotic configurations without an excessive tuning of the parameters or customization. The cerebellum-based control system is indeed able to deal with changing dynamics and interactions with the environment. Important insights regarding the relationship between the bio-inspired control system functioning and the complexity of the task to be performed are obtained.

## 1. Introduction

Controlling a robotic system that operates in an uncertain environment can be a difficult task if the analytical model of the system is not accurate. Models are the most essential tools in robotic control (Francis and Wonham, 1976), however, modeling errors are frequently inevitable in complex robots, for instance humanoids and soft robots. Such redundant modern robots are mechanically complex and often interacts with unstructured dynamical environments (Nakanishi et al., 2008; Nguyen-Tuong et al., 2009). Traditional hand-crafted models and standard physics-based modeling techniques do not sufficiently take into account all the unknown nonlinearities and complexities that these system present. This lack consequentially leads to a reduced tracking accuracy or, in the worst case, to unstable null-space behavior.

Modern autonomous and cognitive robots are requested to adapt not only the decisions but also the forces exerted in any varying condition and environment. The selected movement can not be executed properly if the robot does not adjust the forces according to the changing dynamics. Because of this, modern learning control methods should automatically generate model based on sensor data streams, so that the robot is not a closed entity, but a system that interacts, and evolves through the interaction with a dynamic environment.

In this paper, we intend to design an adaptive learning algorithm to control the movements of a complex nonlinear dynamical system. In particular, we assume that: the Jacobian poorly describes the actual system; the robot interacts with one or more unmodeled external objects; the sensor-actuator system is distributed and not all the states are observable or can be describe with parametric function designed off-line; the action/state space is continuous and high-dimensional. The control system should solve the inverse dynamics control problem of a multiple-joint robotic system affected by static and dynamic external disturbances during the execution of a repeated task. The controller is envisioned to reduce the tracking accuracy of each actuator through force-based control input.

In early days of adaptive self-tuning control, models were learned by fitting open parameters of predefined parametric models (Atkeson et al., 1986; Annaswamy and Narendra, 1989; Wittenmark, 1995; Khalil and Dombre, 2002). Although this method had great success in system identification and adaptive control techniques (Ljung, 2007), the estimation of the open parameters can lead to several problems, such as: slow adaptation; unmodeled behavior and persistent excitation issue (Narendra and Annaswamy, 1987); inconsistency of the estimated physical parameters (Ting et al., 2006); unstable reaction to high estimation error. In recent years, non-parametric approach has been shown to be an efficient tool in the resolution and prevention of the aforementioned problems thanks to the adaptation of the model to the data complexity (Nguyen-Tuong and Peters, 2011), and several methods have been proposed (Farrell and Polycarpou, 2006), such as neural networks (Patino et al., 2002), and statistical methods (Kocijan et al., 2004; Nakanishi and Schaal, 2004; Nakanishi et al., 2005).

In the eighties, Narendra's research group at Yale University exploited the adaptability of artificial neural networks (ANNs) to identify and control nonlinear dynamical systems (Narendra and Mukhopadhyay, 1991a,b, 1997; Narendra and Parthasarathy, 1991). Their experiments showed that the versatility of the ANNs resulted beneficial for controlling the different behaviors that characterize complex dynamical systems. Although the robustness of the classic parametric method in most of the control scenarios, ANNs were largely used in adaptive control to overcome uncertainties, unmodeled nonlinearities and to handle more complex state space systems (Glanz et al., 1991; Sontag, 1992; Zhang et al., 2000; Patino et al., 2002; He et al., 2016, 2018). As matter of fact, the non-linear components and the layered structure that distinguish the ANNs facilitate the mapping and constrain the effects of nonlinearities. Furthermore, the on-line adjustment of the parameters respect to the input-output relationship without any strict structural parameterization results advantageous for adapting to time-dependent changes.

In the Nighties thanks to the extended application of ANNs in robotics, Juyang Weng introduced the Autonomous Mental Development approach (AMD) to artificial intelligence (Weng et al., 1999a; Weng and Hwang, 2006). Weng theories were mainly inspired by how the biological systems efficiently calibrate their movements under internal and environmental changes. Accordingly to AMD the robot have to be embodied in the environment, and its processing is not preprogrammed but is the result of the continuous and real-time interaction within the two systems (Weng et al., 1999b, 2000; Weng, 2002). Respect to classic parametric approaches, the developing artificial agent creates and adapts models describing itself and its relation with the environment rather than learning and estimating parameters of a mathematical model built off-line. These theories found large application for high level cognition tasks (see Vernon et al., 2007 for a review) but were also applied to low level control in visually-guided robots (Metta et al., 1999; Ugur et al., 2015; Luo et al., 2018).

With the aim of mimicking artificially the motor efficiency of the biological system, James S. Albus proposed a neural network-based learning algorithm for robotic controller based on theories of central nervous system (CNS) structure and function: the “cerebellar model articulation controller,” commonly known as CMAC module (Albus, 1972). Several studies in literature demonstrated that, the anatomy and physiology of the cerebellum is suitable for the acquisition, development, storage and use of the internal models describing the interaction within body and environment (Wolpert et al., 1998). Moreover, the cerebellum is composed by separated regions which functionality relies both on the internal structure of the circuit and on the connection with other CNS areas (Houk and Wise, 1995; Caligiore et al., 2017): each region receives both the desired movements from the cortex and the sensory information from tendons, joints and muscles spindles and elaborates a signal that corrects whereas other CNS region are lacking. As matter of fact, subjects affected by cerebellum damage often present motor deficit, such as uncoordinated and ballistic multiple-joint movements (Schmahmann, 2004). For this reason in the last decades, scientists tried to explain the roles of the cerebellum in motor control, especially its contribution to sensory acquisition and timing and its involvement in the prediction of the sensory consequences of action. Moreover, this adaptive control nature motivated several researchers toward a deeper understanding of the cerebellum for robotics application.

Two main research lines born since Marr and Albus proposed the first artificial cerebellum-like network as pattern-classifier for controlling a robotic manipulator (Marr, 1969; Albus, 1972): the first research line focuses on purely industrial application and has as major representative W. Thomas Miller; the second research line, mainly represented by Mitsuo Kawato, deep-rooted in neuroscience and kept investigating on the biological evidence of the cerebellum structure and functionalities in relation to other CNS areas (Kawato et al., 1987; Kawato, 1999).

Miller applied the CMAC module in a closed loop vision-based controller to solve the forward mapping with direct modeling (Miller, 1987). Although the advantages, such as the rapid algorithmic computation based on least-mean-square training and the fast incremental learning, this approach lack of generalization and is sensitive to noise and large error (Miller et al., 1990). Over the years, researchers have been focusing on solving these drawbacks and the CMAC module has been mostly used as non-linear function approximator to boost the tracking accuracy of the adaptive controller and mitigate the effects of the approximation errors (Lin and Chen, 2007; Chen, 2009; Guan et al., 2018; Jiang et al., 2018). Although the promising results obtained by these applications of the CMAC network, this industrial research line did not completely exploit the overall capabilities and components of the cerebellum. It is worthy to note that the CMAC module mimic the cerebellar circuit only at the granular-purkinje level, for this reason only the mapping and classification functionalities are exploited.

The neuroscientific research line has been investigating mainly on the layered structure of the cerebellar circuit proposing several synaptic plasticity models (Luque et al., 2011, 2014, 2016; Casellato et al., 2015; D'Angelo et al., 2016; Antonietti et al., 2017), network models (Chapeau-Blondeau and Chauvet, 1991; Buonomano and Mauk, 1994; Ito, 1997; Mauk and Donegan, 1997; Yamazaki and Tanaka, 2007; Dean et al., 2010), adaptive linear filter model (Fujita, 1982; Barto et al., 1999; Fujiki et al., 2015), and combination of both (Tolu et al., 2012, 2013). These cerebellar-like models were embedded into bio-inspired control architectures to analyze how the cerebellum adjusts the output of the descending motor system of the brain during the generation of movements (Kawato et al., 1987; Ito, 2008), and how it predicts the action, minimizes the sensory discrepancy and cancels the noise (Nowak et al., 2007; Porrill and Dean, 2007). The experiments regarded the generation of voluntary movements with both simulated and real robots, e.g., eye blinking classical conditioning (Antonietti et al., 2017), vestibulo-ocular task (Casellato et al., 2014), the gaze stabilization (Vannucci et al., 2016), and perturbed arm reaching task operating in closed-loop (Garrido Alcazar et al., 2013; Tolu et al., 2013; Luque et al., 2016; Ojeda et al., 2017). From the analysis of the literature, it then emerged that research groups have treated the robots as stand-alone systems without interactions with the environment, while the real world is more complex and every external interaction counts. It is worth mentioning that the previous works have been employed for motor learning and control of simplified objects.

In this paper we present a robotic control architecture to overcome modeling error and to constrain the effects of uncertainties and external disturbances. The proposed controller is composed of a static component based on a classic feedback control methods, and of an adaptive decentralized neural network that mimic the functionality and morphology of the cerebellar circuit. The cerebellar-like module add feed-forward corrective torque to the feedback controller action (Ito, 1984; Miyamoto et al., 1988). A non-parametric nonlinear function approximation algorithm have been employed to map on-line and to reduce the high dimensional and redundant input space. The algorithm creates the internal model describing the interaction within system and environment. This model is kept under development throughout the execution of the task. The neural network mimic the composition of the cerebellar microcircuit. The layered structure of the network constrains the effects of nonlinearities and external perturbations. The network weights are based on non-linear and multidimensional learning rules that mimic the cerebellar synaptic plasticities (Garrido Alcazar et al., 2013; Luque et al., 2014).

This manuscript extends the previous works under three main aspects: 1. cerebellar-like network topology and input data; 2. feedback control-input; 3. dynamic control under external changing conditions. With the aim at giving more insights into the capacity of the cerebellum of generating control terms in the framework of accurate control tasks, the following research questions come naturally to mind: can a control system be generalized to control robotic agents by endowing them with adaptive capabilities? Can accurate and smooth actions in a dynamic environment be performed by the extrapolation of valuable sensory-motor information from heterogeneous dynamical stimuli? Does this sensory-motor information extrapolation facilitate the motor prediction and adaptation in changing conditions? The tests were carried out in the Neuro-robotics Platform (Falotico et al., 2017) with the virtual humanoid robot iCub. The robot arm has to follow a planned movement overcoming the disturbances provoked by a table attached to the hand and a ball running on it. A similar example was solved by employing a conventional control law together with computer vision techniques (Awtar et al., 2002; Levinson et al., 2010). However, this approach assumes a fixed robot morphology defined and described before running the experiment, and there is no run-time adaptation to the “biological changes” as we see in human beings. Balancing a table with a ball running on it is a relevant example of how humans learn to calibrate, coordinate, and adapt their movements, hence, we investigate how robots can achieve this task following the biological approach. Probst et al. (2012) also followed the biological approach; they tackled the problem taking into account the dynamics of the system, four different forces are found by means of a liquid state machine and applied in four different points of the table to achieve the balancing task. A supervised learning rule is used for the training step, which concludes that after 2,500 s no further improvement of the performance is obtained.

Hence, the main advantages of our model are: the low amount of (sometimes implausible) prior information for the control, a fast reactive robotic control system, an on-line self-adaptive learning system. Thanks to these features the robot can perform a determinate physical task and adapt to changing conditions. In conclusion, this approach introduces a fast and flexible control architecture that can be applied to different robotic platforms without any/excessive customization.

In the first section that follows, we present the control architecture, the adopted cerebellar-like model and the description of the method. In the second section, we report the experimental setup as well as the results of the comparison study of four control system approaches including the respective analysis. Finally, we will discuss the main findings of the study correlating them to previous literature.

## 2. Materials and Methods

**Figure 1:**
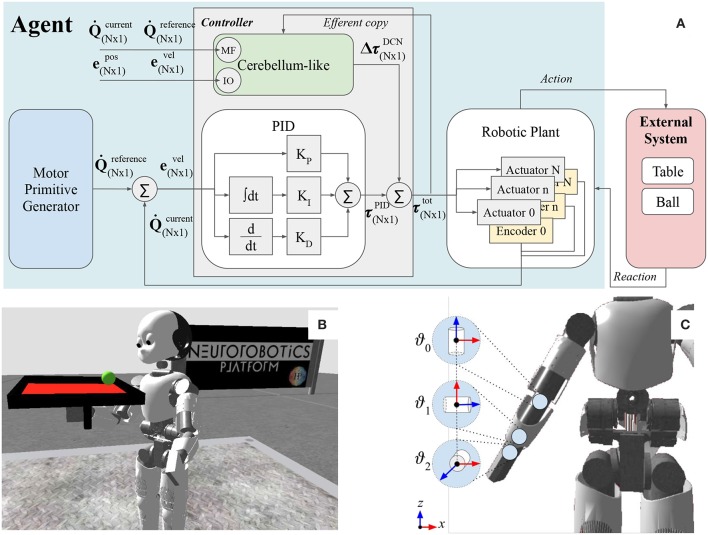
**(A)** The figure illustrates the main components of the functional architecture scheme and the link with the artificial robot agent and the external system. **(B)** The humanoid Icub holding the table-ball system in the simulation environment NRP. **(C)** Three controlled joints: wrist prosup ϑ_0_, wrist yaw ϑ_1_, wrist pitch ϑ_2_.

In this section, we present our bio-inspired approach to solve the problem of controlling the right arm of the ICub humanoid robot despite the occurrence of an external perturbation. The experiment consists of a simulated humanoid robot that executes a requested movement using three controlled joints of the right arm. During the simulation, a ball is launched on the table that is attached to the robot's right hand; the ball is free to roll on the table, as illustrated in Figure 1B. The movements of the ball are provoked by the shaking of the robot arm and consequentially of the table. The key information about the external system components (e.g., the ball and table) are reported in Table 1.

**Table 1:** External system features.

	**Mass **[Kg]****	**Volume **[m^3^]****	**Static friction coefficient**	**Dynamic friction coefficient**
Ball	0.01	6.54 × 10^−5^	0.02	0.01
Table top	0.1	9 × 10^−4^	0.01	0.01

The proposed control architecture (Figure 1A) is composed of three main building blocks: the *robotic plant*, which is the physical structure (section 2.1); the *motor primitive generator*, which is responsible of the trajectory generation (section 2.2); the *controller*, which elaborates the torque commands to move each motor to the desired set point (section 2.3).

### 2.1. Robotic Plant

The Icub humanoid robot is 104 cm tall and it is equipped with a large variety of sensors (such as gyroscopes, accelerometers, F/T sensors, encoders, two digital cameras) and 53 actuated joints that move the waist, head, eyes, legs, arms, and hands. During the experimental tests, eight revolute joints of the right arm were actuated: four joints were kept constant to maintain the arm up (e.g., elbow, shoulder roll, shoulder yaw, and shoulder pitch), and three joints were controlled in effort by the proposed control system (namely wrist prosup, wrist yaw and wrist pitch). The axis orientation of the controlled actuators are illustrated in Figure 1C. Additional information about the actuated joints are reported in Table 2. In this work, we used the encoder to only read the state of the controlled joints (e.g., angular position, and velocity) and save it in the process variables,

(1) $$\begin{array}{l} {\text{Q}}_{N\times 1}^{c}\left(t\right)=\left[\begin{array}{c} \vartheta_{c,0}\left(t\right)\\ \ldots \\ \vartheta_{c,N}\left(t\right) \end{array}\right]\;where\;N=2, \end{array}$$

(2) $$\begin{array}{l} {\overset{\cdot }{\text{Q}}}_{N\times 1}^{c}\left(t\right)=\left[\begin{array}{c} {\overset{\cdot }{\vartheta }}_{c,0}\left(t\right)\\ \ldots \\ {\overset{\cdot }{\vartheta }}_{c,N}\left(t\right) \end{array}\right]\;where\;N=2, \end{array}$$

**Table 2:** Actuated joints information: the wrist actuators (highlighted in yellow) are controlled in effort while the elbow and shoulder motors are kept to a constant angular position.

	**Min ϑ[rad]**	**Max ϑ[rad]**	**Max $\dot{\vartheta }$ [rad · sec^−1^]**	**Control**	**Value**
Wrist prosup *n* = 0	–0.8726	0.8726	100	Effort	Controlled variable
Wrist yaw *n* = 1	–0.4363	0.4363	100	Effort	Controlled variable
Wrist pitch *n* = 2	–1.1344	0.1745	100	Effort	Controlled variable
Elbow	0.0959	1.8500	100	Position	Constant = 1.14 [rad]
Shoulder roll	0.0000	2.80649	100	Position	Constant = 0.1 [rad]
Shoulder yaw	–0.645772	1.74533	100	Position	Constant = –0.1 [rad]
Shoulder pitch	–1.65806	0.0872665	100	Position	Constant = –0.9 [rad]

### 2.2. Motor Primitive Generator

The motor primitive generator plans the trajectory for each actuated joint and communicates the reference value to the control system at each time step. The reference angular position and velocity of each joint are defined as oscillators with fixed amplitude, natural frequency and phase,

(3) $$\begin{array}{l} {\text{Q}}_{N\times 1}^{r}\left(t\right)=\left[\begin{array}{c} \vartheta_{r,0}\left(t\right)\\ \ldots \\ \vartheta_{r,N}\left(t\right) \end{array}\right]=\left[\begin{array}{c} A_{0}\cdot \text{sin}\left(2\pi \text{f}t+\varphi_{0}\right)\\ \ldots \\ A_{N}\cdot \text{sin}\left(2\pi \text{f}t+\varphi_{N}\right) \end{array}\right], \end{array}$$

(4) $$\begin{array}{l} {\overset{\cdot }{\text{Q}}}_{N\times 1}^{r}\left(t\right)=\left[\begin{array}{c} {\overset{\cdot }{\vartheta }}_{r,0}\left(t\right)\\ \ldots \\ {\overset{\cdot }{\vartheta }}_{r,N}\left(t\right) \end{array}\right]=\left[\begin{array}{c} 2\pi \text{f}A_{0}\cdot \text{cos}\left(2\pi \text{f}t+\varphi_{0}\right)\\ \ldots \\ 2\pi \text{f}A_{N}\cdot \text{cos}\left(2\pi \text{f}t+\varphi_{N}\right) \end{array}\right], \end{array}$$

where *N* = 2. The temporal frequency is *f* = 0.25Hz, while the oscillations **A** amplitude and φ phase of each joint are set to:

$$\begin{array}{l} {\text{A}}_{1\times N}=\left[\begin{array}{c} A_{0},\;A_{1},\;A_{2} \end{array}\right]=\left[\begin{array}{c} 0.1727,\;0.1363,\;0.0345 \end{array}\right]\;\text{rad}\\ \varphi_{1\times N}=\left[\begin{array}{c} \varphi_{0},\;\varphi_{1},\;\varphi_{2} \end{array}\right]=\left[\begin{array}{c} 0.5\pi ,\;0.5\pi ,\;0.0 \end{array}\right]\;\text{rad}. \end{array}$$

### 2.3. Controller

The *controller* block (Figure 1A) is composed of a static component based on classic control methods (section 2.3.1), and of an adaptive decentralized block representing the bio-inspired regulator, i.e., the cerebellar-like circuit (section 2.3.2). Both sub-blocks receive information about the **Q**^*c*^, ${\overset{\cdot }{\text{Q}}}^{c}$ process variables measured from the encoders located in the *robotic plant* (Equations 1, 2), and the **Q**^*r*^,${\overset{\cdot }{\text{Q}}}^{r}$ reference trajectory signals from the *motor primitive generator* (Equations 3, 4). The controller directly sends the τ^*tot*^ total control input to the robot servo controller which actuates the joints for δ*t* = 0.5*s*. The τ^*tot*^ total control input is expressed as the result of a feed-forward compensation (as the AFEL architecture proposed by Tolu et al., 2012),

(5) $$\begin{array}{l} \tau_{N\times 1}^{tot}=\left[\begin{array}{c} \tau_{0}^{tot}\\ \ldots \\ \tau_{N}^{tot} \end{array}\right]=\left[\begin{array}{c} \tau_{0}^{PID}+\Delta \tau_{0}^{DCN}\\ \ldots \\ \tau_{N}^{PID}+\Delta \tau_{N}^{DCN} \end{array}\right], \end{array}$$

τ^*tot*^ where $\tau_{n}^{PID}$ and $\Delta \tau_{n}^{DCN}$ (where *n* = 0, …, *N*) are the contributions from the static and the adaptive bio-inspired controller respectively.

#### 2.3.1. Feedback Controller

The static control system refers to the classic feedback control scheme with PID regulator. It is defined static due to its time-constant control terms. The closed-loop system continuously computes the ${\text{e}}_{{\overset{\cdot }{\vartheta }}_{n}}$ angular velocity error of each joint as the difference between the ${\overset{\cdot }{\vartheta }}_{r,n}$ reference (Equation 4) and the ${\overset{\cdot }{\vartheta }}_{c,n}$ process variable (Equation 2),

(6) $$\begin{array}{l} {\text{e}}_{N\times 1}^{vel}=\left[\begin{array}{c} e_{{\overset{\cdot }{\vartheta }}_{0}}\\ \ldots \\ e_{{\overset{\cdot }{\vartheta }}_{N}} \end{array}\right]=\left[\begin{array}{c} {\overset{\cdot }{\vartheta }}_{r,0}-{\overset{\cdot }{\vartheta }}_{c,0}\\ \ldots \\ {\overset{\cdot }{\vartheta }}_{r,N}-{\overset{\cdot }{\vartheta }}_{c,N} \end{array}\right]. \end{array}$$

The ${\text{e}}_{{\overset{\cdot }{\vartheta }}_{n}}$ error (where *n* = 0, …, *N*) is used to apply correction to each controlled joint in terms of effort,

(7) $$\begin{array}{l} \tau_{N\times 1}^{PID}={\left[\begin{array}{c} \tau_{0}^{PID},\;\ldots \;,\;\tau_{N}^{PID} \end{array}\right]}^{T}, \end{array}$$

according to the independent joint control law expressed as:

(8) $$\begin{array}{l} \tau_{n}^{PID}\left(t\right)=K_{P,n}\cdot e_{{\overset{\cdot }{\vartheta }}_{n}}+K_{I,n}\cdot \int_{t-\Delta t}^{t}e_{{\overset{\cdot }{\vartheta }}_{n}}\left(t^{\prime }\right)dt^{\prime }+K_{D,n}\cdot \frac{\text{d}{\overset{\cdot }{\vartheta }}_{n}\left(t\right)}{\text{d}t}\;\\ \;for\;n=0,\;\ldots \;,N\;,\; \end{array}$$

where the integration time window is Δ*t* = 10 samples. The regulator is tuned to weakly operate in a linearized condition which excludes the presence and disturbance of the ball, hence the proportional, integrative and derivative terms are static and set respectively to,

$$\begin{array}{l} {\text{K}}_{P}=\left[\begin{array}{c} K_{P,0},\;K_{P,1},\;K_{P,2} \end{array}\right]=\left[\begin{array}{c} 2.9000,\;2.3000,\;2.3500 \end{array}\right]\\ {\text{K}}_{I}=\left[\begin{array}{c} K_{I,0},\;K_{I,1},\;K_{I,2} \end{array}\right]=\left[\begin{array}{c} 1.9400,\;1.9000,\;1.9000 \end{array}\right]\\ {\text{K}}_{D}=\left[\begin{array}{c} K_{D,0},\;K_{D,1},\;K_{D,2} \end{array}\right]=\left[\begin{array}{c} 0.0050,\;0.0001,\;0.0004 \end{array}\right]. \end{array}$$

#### 2.3.2. Cerebellar-Like Model

**Figure 2:**
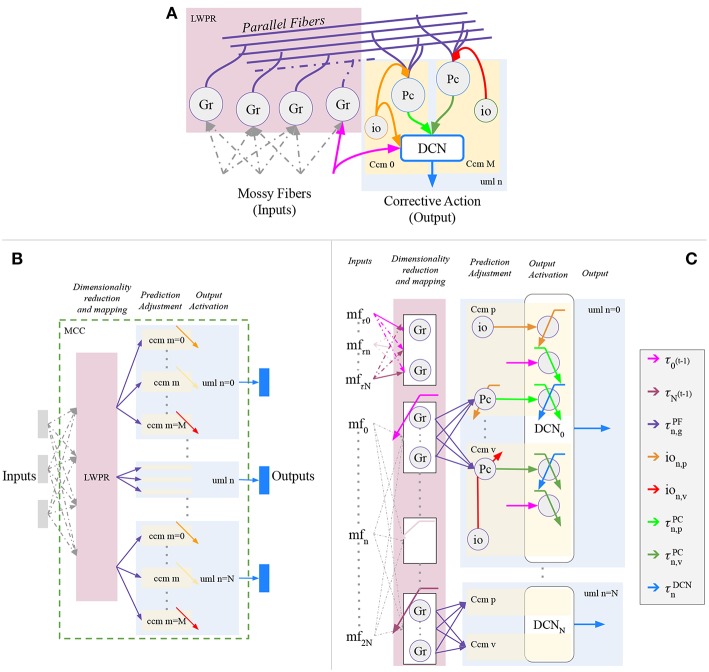
Proposed cerebellar-like circuit in analogy with D'Angelo et al. (2016). **(A)** canonical micro-circuit. Proposed cerebellar-like neural network **(B)** structural partition and **(C)** details.

The proposed cerebellar-like network has been designed to solve robotic problems (Figure 2). In particular, the sensory input and the corrective action in output refer to entities regarding the actuated motors, such as motor angular position, velocity or effort. Electrophysiological evidence about the encoding of movement kinematics has been found at all levels of the cerebellum; for example, in this review (Ebner et al., 2011), reported that the mossy fibers (MF) inputs encode the position, direction, and velocity of limb movements. Moreover, many hypotheses suggest that the cerebellum directly contributes to the motor command required to produce a movement. In our model, the input-output relationship is based on the previous suggestions and the signal propagation throughout the cerebellar network layers is in accordance with the robotic control application. The main design concept is that the signal propagating inside the circuit have the same dimension of the Δτ^*DCN*^ output signal from the Deep Cerebellar Nuclei (DCN). The propagated signal is modulated inside the network by other signals that are correlated with the intrinsic features of the controlled plant, such as position and velocity terms, in order to have a complete description of the state.

The neural network structure is divided into separated modules (Figure 2B), or namely *Unit Learning Machine* (uml) (Tolu et al., 2012, 2013). Assuming that the robot plant is composed by N controllable object, then each uml is specialized on the n-th controlled object (where *n* = 0, …, *N*), or rather the DCN output of the uml will be the cerebellar contribution for the specific object. The uml itself is separated into M sub-modules which represent the *canonical cerebellar microcircuit* (ccm). Each ccm is specialized with respect to a specific feature describing the behavior of the n-th controlled object. The overall umls and other structures, that are dedicated to the dimensionality reduction and mapping of the sensory information, compose together the *Modular Cerebellar Circuit* (MCC).

In the proposed experiment, the canonical cerebellar microcircuits (ccm) of each controlled object are specialized in *p* position and in *v* velocity. In details, the Purkinje layer of each *n*−th uml presents a pair of Purkinje cells (PC) (Figure 2C), specialized in position *Pc*_*n, p*_ and velocity *Pc*_*n, v*_ respectively through different climbing fibers (*io*_*n, p*_, and *io*_*n, v*_). Moreover, the bio-inspired controller receives the same sensory information of the feedback controller (section 2.3.1), but it is intended to correct the *e*_ϑ_*n*__ angular position error, whereas the PID corrects the $e_{{\overset{\cdot }{\vartheta }}_{n}}$ angular velocity error. This is solved through the connection inferior olive-deep cerebellar nuclei (IO-DCN), which conveys information about the angular position error. An additional aspect, the inferior olive signals differs from Kawato's feedback error learning theory (Kawato, 1990) and our previous experiments (Tolu et al., 2012, 2013), because the Jacobian does not correctly approximate the system, therefore the required conditions are not satisfied and it is not efficient to compare the motor signals.

The mossy fibers transmit the information about the current and reference state of the controlled joints in terms of angular velocity to the granular cells (Gr),

(9) $$\begin{array}{l} {\text{MF}}_{2N\times 1}\left(t\right)=\left[\begin{array}{c} mf_{0}\left(t\right)\\ \ldots \\ mf_{2N}\left(t\right) \end{array}\right]=\left[\begin{array}{c} {\overset{\cdot }{\text{Q}}}_{N\times 1}^{r}\left(t\right)\\ {\overset{\cdot }{\text{Q}}}_{N\times 1}^{c}\left(t\right) \end{array}\right]=\left[\begin{array}{c} {\overset{\cdot }{\vartheta }}_{r,0}\left(t\right)\\ \ldots \\ {\overset{\cdot }{\vartheta }}_{r,N}\left(t\right)\\ {\overset{\cdot }{\vartheta }}_{c,0}\left(t\right)\\ \ldots \\ {\overset{\cdot }{\vartheta }}_{c,N}\left(t\right) \end{array}\right]. \end{array}$$

The granular layer-parallel fibers network is the circuit area committed to the mapping of the mossy fibers signals and to the prediction of the next output given the current sensory input (Marr, 1969; Albus, 1971). As in our previous works (Tolu et al., 2012, 2013), we artificially represented this network with the Locally Weighted Projection Regression algorithm (LWPR) (Vijayakumar and Schaal, 2000). The LWPR resulted an efficient method for the fast on-line approximation of non-linear functions in high dimensional spaces. Given the **MF**(*t*) mossy fibers input vector (Equation 9), the LWPR creates G local linear models that in our scheme represent the *Gr*_*g*_ granular cells (for *g* = 0, …, *G*). Each linear model employs the **MF**(*t*) to make a ${\hat{\tau }}_{n,g}^{gr}\left(t\right)$ prediction of the control input $\tau_{n}^{tot}\left(t-1\right)$ (where n=1,…,N). The total output of the granular-parallel fibers network is the weighted mean of all the linear models specialized in velocity,

(10) $$\begin{array}{l} {\hat{\tau }}_{n}^{PF}\left(t\right)=\frac{\sum_{g=1}^{g=G}w_{n,g}^{gr}\left(t\right)\cdot {\hat{\tau }}_{n,g}^{gr}\left(t\right)}{\sum_{g=1}^{g=G}w_{n,g}^{gr}\left(t\right)}\;for\;n=1,\ldots ,N, \end{array}$$

where $w_{n,g}^{gr}$ and ${\hat{\tau }}_{n,g}^{gr}$ are defined in Vijayakumar and Schaal (2000).

In our scheme, there are two Purkinje cells per controlled joint *Pc*_*n, p*_ and *Pc*_*n, v*_ (where *n* = 0, …, *N*). The $w_{n,p}^{pf-pc}$^1^ synapses connecting the parallel fibers and the *Pc*_*n, p*_ (PF-PC connection) (Garrido Alcazar et al., 2013), are modulated by the *io*_*n, p*_ inferior olive (IO) signal,

(11) $$\begin{array}{l} io_{n,p}\left(t\right)={ẽ}_{\vartheta_{n}}\left(t\right), \end{array}$$

that transmits the information about the ẽ_ϑ_*n*__ normalized angular position error of the *n*−th joint,

(12) $$\begin{array}{l} e_{\vartheta_{n}}\left(t\right)=\vartheta_{r,n}\left(t\right)-\vartheta_{c,n}\left(t\right), \end{array}$$

while the $w_{n,v}^{pf-pc}$ ^1^ synaptic strengths between the parallel fibers and the *Pc*_*n, v*_, are modulated by the *io*_*n, v*_ inferior olive signal,

(13) $$\begin{array}{l} io_{n,v}\left(t\right)={ẽ}_{{\overset{\cdot }{\vartheta }}_{n}}\left(t\right), \end{array}$$

that transmits the information about the ${ẽ}_{{\overset{\cdot }{\vartheta }}_{n}}$ normalized angular velocity error of the *n*−th joint (Equation 6). The $w^{pf-pc}\left(t,io_{0}\left(t\right)\right)$ weighting kernel tends to support the control actions that lead to an error lower than a specific threshold *e*^*thresh*^,

(14) $$\begin{array}{lll} {\text{e}}_{\vartheta }^{thresh,pc} & = & \left[\begin{array}{c} e_{\vartheta_{0}}^{thresh,pc}\\ \ldots \\ e_{\vartheta_{N}}^{thresh,pc} \end{array}\right]=\left[\begin{array}{c} w_{0,p}^{pf-pc}\left(t,io_{0}^{p}\left(t\right)=0\right)\cdot max\left(e_{\vartheta_{0}}\right)\\ \ldots \\ w_{N,p}^{pf-pc}\left(t,io_{N,p}\left(t\right)=0\right)\cdot max\left(e_{\vartheta_{N}}\right) \end{array}\right]\\ \; & = & \left[\begin{array}{c} 0.012\\ 0.008\\ 0.002 \end{array}\right]\left[\text{rad}\right], \end{array}$$

(15) $$\begin{array}{lll} {\text{e}}_{\overset{\cdot }{\vartheta }}^{thresh,pc} & = & \left[\begin{array}{c} e_{{\overset{\cdot }{\vartheta }}_{0}}^{thresh,pc}\\ \ldots \\ e_{{\overset{\cdot }{\vartheta }}_{N}}^{thresh,pc} \end{array}\right]=\left[\begin{array}{c} w_{0,v}^{pf-pc}\left(t,io_{0}^{v}\left(t\right)=0\right)\cdot max\left(e_{{\overset{\cdot }{\vartheta }}_{0}}\right)\\ \ldots \\ w_{N,v}^{pf-pc}\left(t,io_{N,v}\left(t\right)=0\right)\cdot max\left(e_{{\overset{\cdot }{\vartheta }}_{N}}\right) \end{array}\right]\\ \; & = & \left[\begin{array}{c} 0.012\\ 0.008\\ 0.002 \end{array}\right]\left[\text{rad}\cdot {\text{sec}}^{-1}\right]. \end{array}$$

Respect to our previous work (Tolu et al., 2012, 2013) the output signals of the Purkinje cells are directly function of the ${\hat{\tau }}_{n}^{PF}\left(t\right)$ prediction instead of the $w_{n,g}^{gr}$ weights,

(16) $$\begin{array}{l} \tau_{n,p}^{PC}\left(t\right)=w_{n,p}^{pf-pc}\left(t,io_{n,p}\left(t\right)\right)\cdot {\hat{\tau }}_{n}^{PF}\left(t\right) \end{array}$$

(17) $$\begin{array}{l} \tau_{n,v}^{PC}\left(t\right)=w_{n,v}^{pf-pc}\left(t,io_{n,v}\left(t\right)\right)\cdot {\hat{\tau }}_{n}^{PF}\left(t\right). \end{array}$$

Afterwards, the $\tau_{n,p}^{PC}\left(t\right)$ $\tau_{n,v}^{PC}\left(t\right)$ Purkinje cells signals are scaled by the synaptic weights $w_{n,p}^{pc-dcn}$ and $w_{n,v}^{pc-dcn}$ ^2^ (Garrido Alcazar et al., 2013), that are modulated by the Purkinje cells and the deep cerebellar nuclei activities (PC-DCN),

(18) $$\begin{array}{l} w_{n,p}^{pc-dcn}=f\left(t,\tau_{n,p}^{PC}\left(t\right),\Delta \tau_{n}^{DCN}\left(t-1\right)\right), \end{array}$$

(19) $$\begin{array}{l} w_{n,v}^{pc-dcn}=f\left(t,\tau_{n,v}^{PC}\left(t\right),\Delta \tau_{n}^{DCN}\left(t-1\right)\right). \end{array}$$

resulting in the input signals,

(20) $$\begin{array}{l} \tau_{n,p}^{PC-DCN}\left(t\right)=w_{n,p}^{pc-dcn}\cdot \tau_{n,p}^{PC}\left(t\right) \end{array}$$

(21) $$\begin{array}{l} \tau_{n,v}^{PC-DCN}\left(t\right)=w_{n,v}^{pc-dcn}\cdot \tau_{n,v}^{PC}\left(t\right). \end{array}$$

In addition, the deep cerebellar nuclei receives the input signals $\tau_{n,p}^{MF-DCN}$, $\tau_{n,v}^{MF-DCN}$ from the mossy fibers and $\tau_{n,p}^{IO-DCN}$ from the inferior olive. In our proposed circuit, the mossy fibers connected to the deep cerebellar nuclei (MF-DCN) conveys the information about the $\tau_{n}^{tot}\left(t-1\right)$ last control input sent to each controlled joint (Equation 5). This input is scaled by the synaptic weights $w_{n,p}^{mf-dcn}$ and $w_{n,v}^{mf-dcn}$ ^3^ (Garrido Alcazar et al., 2013), modulated by the respective *n*−th Purkinje cells activities,

(22) $$\begin{array}{l} \tau_{n,p}^{MF-DCN}\left(t\right)=w_{n,p}^{mf-dcn}\left(t,\tau_{n,p}^{PC}\left(t\right)\right)\cdot \tau_{n}^{tot}\left(t-1\right), \end{array}$$

(23) $$\begin{array}{l} \tau_{n,v}^{MF-DCN}\left(t\right)=w_{n,v}^{mf-dcn}\left(t,\tau_{n,v}^{PC}\left(t\right)\right)\cdot \tau_{n}^{tot}\left(t-1\right). \end{array}$$

The $\tau_{n,p}^{IO-DCN}$ inferior olive contribution in the deep cerebellar nuclei (IO-DCN) is given by the *io*_*n, p*_ (Equation 11), which is modulated by the $w_{n,p}^{io-dcn}$ ^4^ synaptic weight (Luque et al., 2014),

(24) $$\begin{array}{l} \tau_{n,p}^{IO-DCN}=w_{n,p}^{io-dcn}\left(t,io_{n,p}\left(t\right)\right)\cdot io_{n,p}\left(t\right). \end{array}$$

The final $\Delta \tau_{n}^{DCN}$ cerebellar corrective term is the result of the $\tau_{n}^{MF-DCN}$ modulated control input subtracted by the $\tau_{n}^{PC-DCN}$ prediction modulated by the current error together with the $\tau_{n,p}^{IO-DCN}$ modulated contribution of the error itself,

(25) $$\begin{array}{lll} \Delta \tau_{n}^{DCN} & = & \left(\tau_{n,p}^{MF-DCN}+\tau_{n,v}^{MF-DCN}\right)-\left(\tau_{n,p}^{PC-DCN}+\tau_{n,v}^{PC-DCN}\right)\\ \; & + & \tau_{n,p}^{IO-DCN}, \end{array}$$

or rather,

$$\begin{array}{l} \begin{array}{ccc} \Delta \tau_{n}^{DCN} & = & \left(\tau_{n}\left(\vartheta_{n},\tau^{tot}\right)+\tau_{n}\left({\overset{\cdot }{\vartheta }}_{n},\tau^{tot}\right)\right)-\left({\hat{\tau }}_{n}^{tot}\left(e_{\vartheta_{n}}\right)+{\hat{\tau }}_{n}^{tot}\left(e_{\overset{\cdot }{\vartheta }}\right)\right)\\ \; & + & \tau_{n}\left(e_{\vartheta_{n}}\right). \end{array} \end{array}$$

### 2.4. Proposed Experiments and Performance Measures

**Figure 3:**
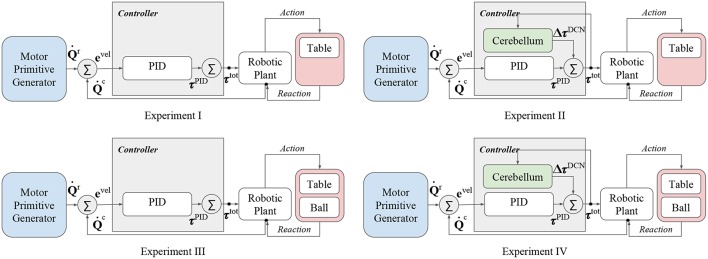
Functional architectures representing the proposed experiments.

The proposed control scheme has been applied in four different experiments with the aim at analyzing the advantages of the bio-inspired controller in presence of dynamical disturbances. In details, the four experiments differ from the presence of the ball and the cerebellar-like controller contribution (Figure 3):

- **Experiment I:** control input without both cerebellum contribution and ball disturbance,
  (26) $$\begin{array}{l} \tau^{tot}=\tau^{PID}\;\left(no\;ball\right); \end{array}$$
- **Experiment II:** control input with cerebellum contribution, without ball disturbance,
  (27) $$\begin{array}{l} \tau^{tot}=\tau^{PID}+\Delta \tau^{DCN}\;\left(no\;ball\right); \end{array}$$
- **Experiment III:** control input without cerebellum contribution, with ball disturbance,
  (28) $$\begin{array}{l} \tau^{tot}=\tau^{PID}\;\left(ball\right); \end{array}$$
- **Experiment IV:** control input with both cerebellum contribution and ball disturbance,
  (29) $$\begin{array}{l} \tau^{tot}=\tau^{PID}+\Delta \tau^{DCN}\;\left(ball\right). \end{array}$$

The performance of each experiment will be measured by analysis of the *mean absolute error* (MAE) evolution computed for the angular position error of each controlled joint (Equation 12),

(30) $$\begin{array}{l} mae_{\vartheta_{n}}\left(k\right)=\frac{\sum_{i=t}^{t+T}|e_{\vartheta_{n}}\left(i\right)|}{T}\;for\;n=0,\ldots ,N. \end{array}$$

The MAE is computed for every trajectory period *T* = 8 s (Equation 3).

## 3. Results

The software describing the system is based on the ROS (Quigley et al., 2009) messaging architecture and is integrated in the Neurorobotics Platform (NRP) (Falotico et al., 2017). The NRP is a simulation environment based on ROS and Gazebo (Koenig and Howard, 2004) which includes a variety of robots, environments and a detailed physics simulator. The three wrist motors are controlled in effort through the Gazebo service *ApplyJointEffort*, while the elbow and the three shoulder motors are controlled in position through their specific ROS topic. The sensory information from the encoders are received with a sampling frequency of *f*_*sampl*_ = 50 Hz. The computer used for the test has the *Ubuntu* 16.04 Operating system (OS type 64−*bit*), the Intel Core™ *i*7 − 7700*HQ* CPU@2.80GHz × 8 processor, and the *GeForce GTX* 1050/*PCIe*/*SSE*2 graphics card.

Each experiment was performed 20 times with a total duration of about 3 min. The recorded data was saved in.csv files and processed for the analysis. The results are expressed as mean value of the 20 tests, and σ standard deviation or 95% confidence interval. In each experiment, the cerebellar-like circuit is activated after *t* = 40 s (or 10*th* iteration), which is the moment all the actuated joints reach a stable configuration (included the shoulder joints and the elbow). In experiments II and IV, the ball is launched on the table after *t* = 5 s (purple vertical line in the figures).

**Figure 4:**
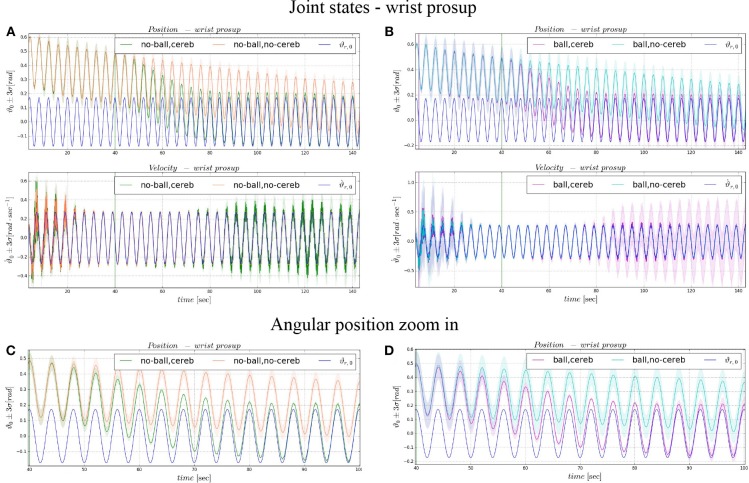
Angular position and velocity wrist prosup: comparison experiment I and II **(A)**, with zoom on the angular position **(C)**; comparison experiment I and II **(B)**, with zoom on the angular position **(D)**. The plots show the results of the 20 tests in terms of mean value (solid line) and 95% confidence interval (colored area). The vertical green line indicates the moment in which the cerebellar-like controller starts giving the corrective action (*t* = 40s). The vertical purple line indicates the instant the ball is launched on the table (*t* = 5s).

**Figure 5:**
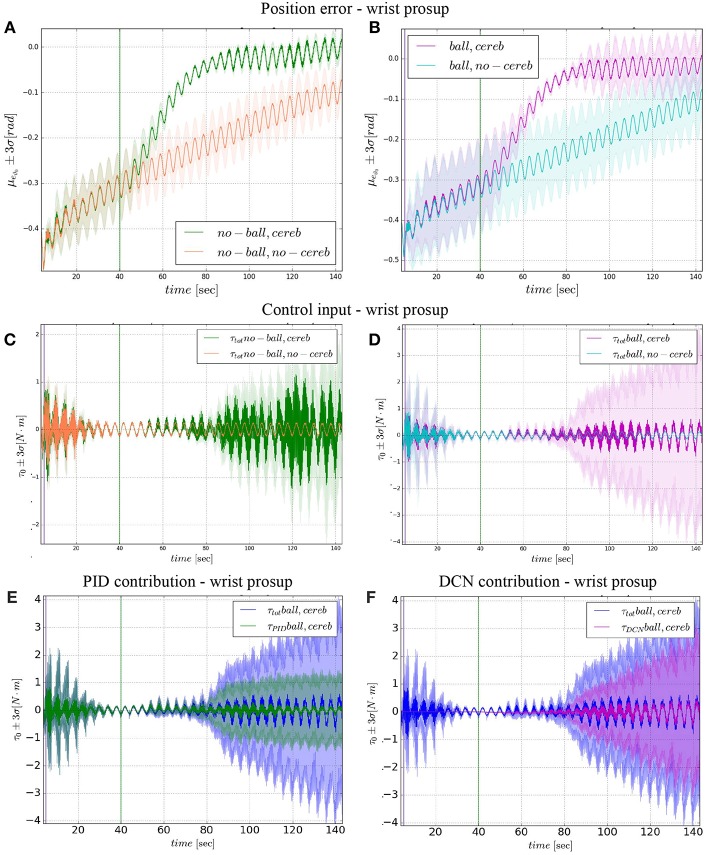
Wrist prosup experimental results. Resulting angular position error *e*_ϑ_0__, comparison experiments I and II **(A)**, comparison experiments III IV **(B)**. Control input $\tau_{0}^{tot}$ evolution, comparison experiments I and II **(C)**, comparison experiments III IV **(D)**. Control input contributions in experiment IV comparisons between: $\tau_{0}^{tot}$ and $\tau_{0}^{PID}$ **(E)**; $\tau_{0}^{tot}$ and $\tau_{0}^{DCN}$ **(F)**. The plots show the results of the 20 tests in terms of mean value (solid line) and 95% confidence interval (colored area). The vertical green line indicates the moment the cerebellar-like controller starts giving the corrective action (*t* = 40s). The vertical purple line indicates the instant the ball is launched on the table (*t* = 5s).

**Figure 6:**
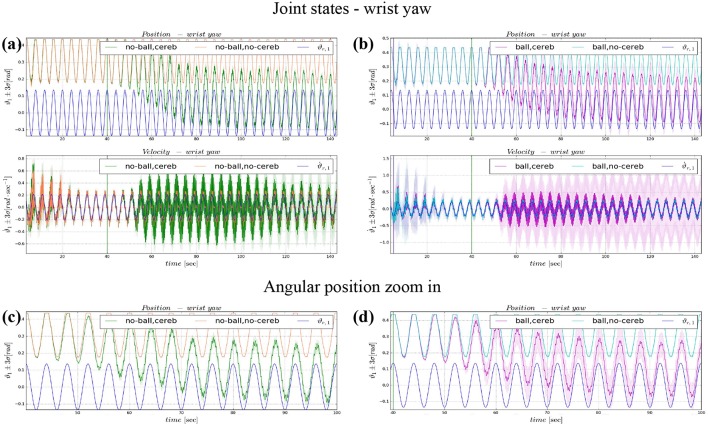
Angular position and velocity wrist yaw: comparison experiment I and II **(a)**, with zoom on the angular position **(c)**; comparison experiment I and II **(b)**, with zoom on the angular position **(d)**. The plots show the results of the 20 tests in terms of mean value (solid line) and 95% confidence interval (colored area). The vertical green line indicates the moment the cerebellar-like controller starts giving the corrective action (*t* = 40s). The vertical purple line indicates the instant the ball is launched on the table (*t* = 5s).

**Figure 7:**
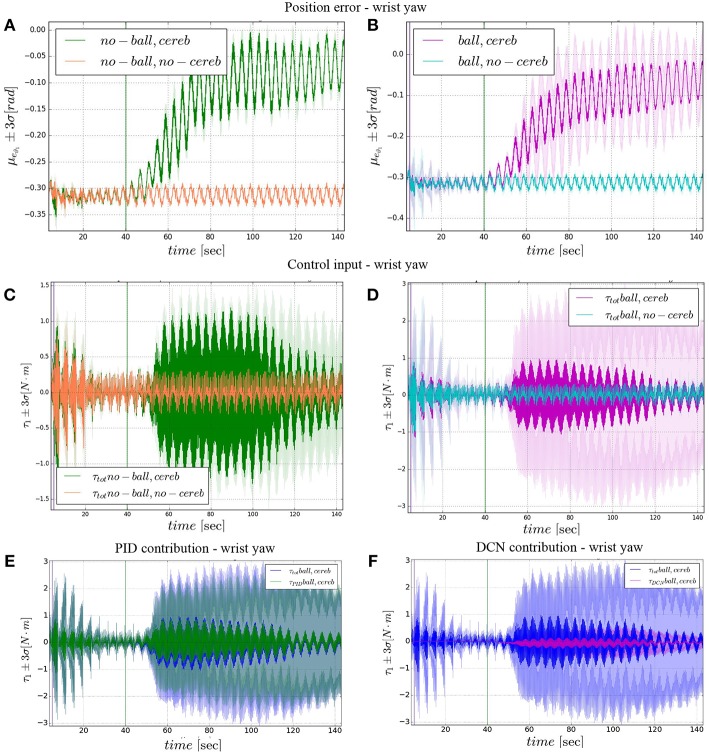
Wrist yaw experimental results. Resulting angular position error *e*_ϑ_1__, comparison experiments I and II **(A)**, comparison experiments III IV **(B)**. The $\tau_{1}^{tot}$ control input evolution, comparison experiments I and II **(C)**, comparison experiments III IV **(D)**. Control input contributions in experiment IV comparisons between: $\tau_{1}^{tot}$ and $\tau_{1}^{PID}$ **(E)**; $\tau_{1}^{tot}$ and $\tau_{1}^{DCN}$ **(F)**.The plots show the results of the 20 tests in terms of mean value (solid line) and 95% confidence interval (colored area). The vertical green line indicates the moment the cerebellar-like controller starts providing the corrective action (*t* = 40s). The vertical purple line indicates the instant the ball is launched on the table (*t* = 5s).

**Figure 8:**
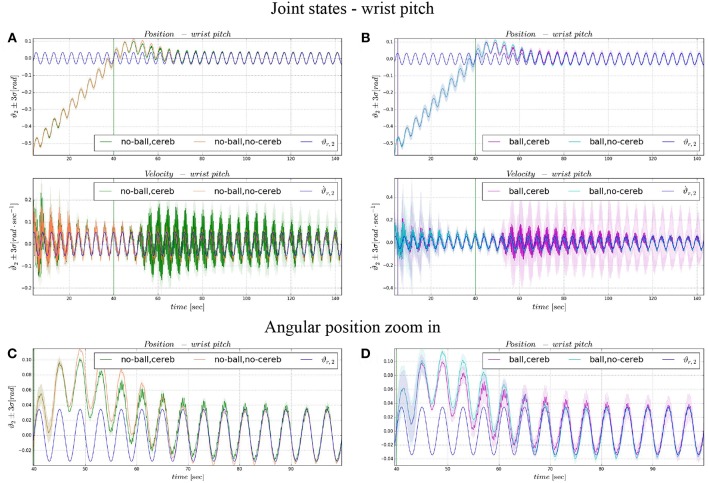
Angular position and velocity wrist pitch: comparison experiment I and II **(A)**, with zoom on the angular position **(C)**; comparison experiment I and II **(B)**, with zoom on the angular position **(D)**. The plots show the results of the 20 tests in terms of mean value (solid line) and 95% confidence interval (colored area). The vertical green line indicates the moment the cerebellar-like controller starts providing the corrective action (*t* = 40s). The vertical purple line indicates the instant the ball is launched on the table (*t* = 5s).

**Figure 9:**
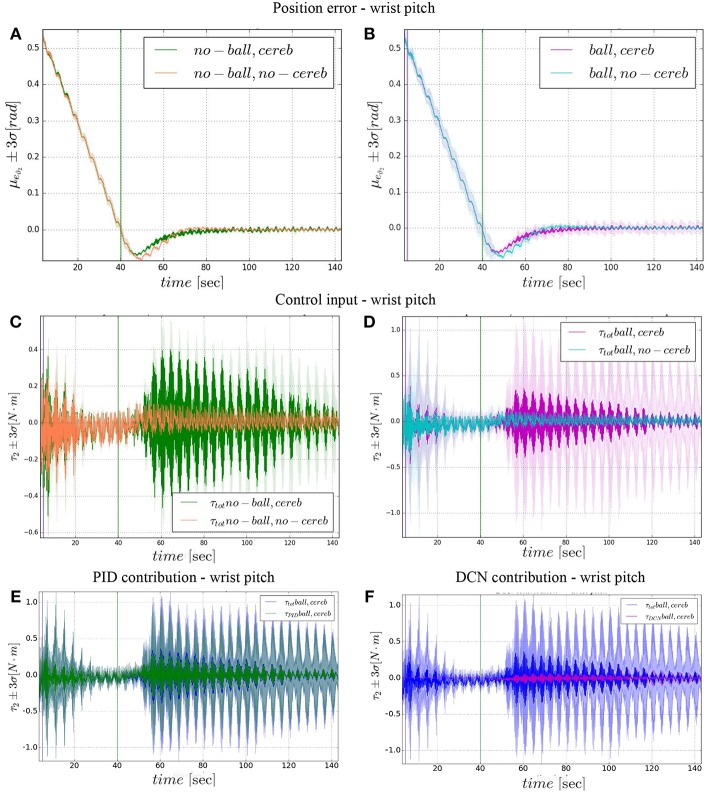
Wrist pitch experimental results. Resulting angular position error *e*_ϑ_2__, comparison experiments I and II **(A)**, comparison experiments III IV **(B)**. The $\tau_{2}^{tot}$ control input evolution, comparison experiments I and II **(C)**, comparison experiments III IV **(D)**. Control input contributions in experiment IV comparisons between: $\tau_{2}^{tot}$ and $\tau_{2}^{PID}$ **(E)**; $\tau_{2}^{tot}$ and $\tau_{2}^{DCN}$ **(F)**. The plots show the results of the 20 tests in terms of mean value (solid line) and 95% confidence interval (colored area). The vertical green line indicates the moment the cerebellar-like controller starts providing the corrective action (*t* = 40s). The vertical purple line indicates the instant the ball is launched on the table (*t* = 5s).

**Figure 10:**
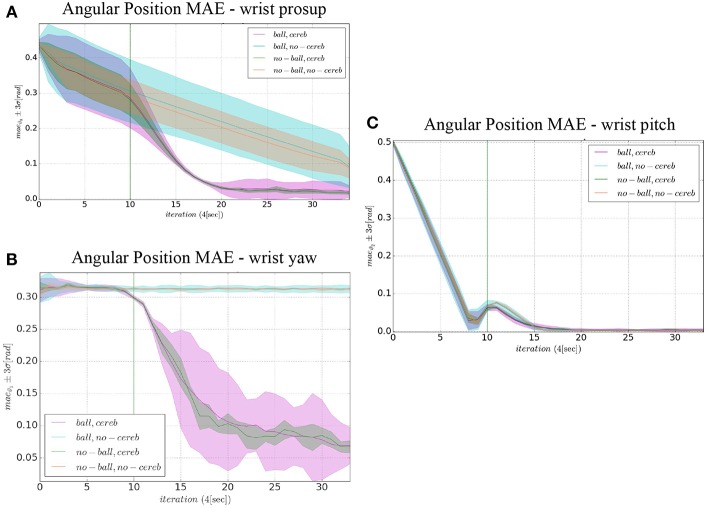
Comparison of the angular position MAE: **(A)** wrist prosup, **(B)** wrist yaw and **(C)** wrist pitch. The plots show the results of the 20 tests in terms of mean value (solid line) and 95% confidence interval (colored area). The vertical green line indicates the moment the cerebellar-like controller starts providing the corrective action (*t* = 40s or *iteration* = 10).

The comparison of the 4 experiments for each controlled joint are presented separately in 3 parts. In each part, we analyze the joint states, i.e., ϑ_*c, n*_(*t*) angular position and ${\overset{\cdot }{\vartheta }}_{c,n}\left(t\right)$ velocity (Figures 4, **6**, **8**), respect to the control action (Figures 5, **7**, **9**). Moreover, we compared the mean absolute error MAE to measure the performance of the different cases (as reported in Table 3 and illustrated in **Figure 10**).

**Table 3:** The mean absolute error (MAE) of the initial and final period (*T* = 4 s).

		**Ball**				**No ball**			
		**Cerebellum**		**No cerebellum**		**Cerebellum**		**No cerebellum**	
		**Initial**	**Final**	**Initial**	**Final**	**Initial**	**Final**	**Initial**	**Final**
*mae*_ϑ_0__ [rad]	μ	0.4136	0.0241	0.4148	0.1104	0.4068	0.0188	0.4136	0.1037
	σ	0.0181	0.0085	0.0273	0.0242	0.0120	0.0017	0.0319	0.0075
*mae*_ϑ_1__ [rad]	μ	0.3148	0.0689	0.3168	0.3123	0.3139	0.0671	0.3172	0.3130
	σ	0.0048	0.0093	0.0070	0.0018	0.0012	0.0031	0.0016	0.0005
*mae*_ϑ_2__ [rad]	μ	0.4380	0.0042	0.4395	0.0019	0.4437	0.0037	0.4401	0.0020
	σ	0.0045	0.0012	0.0063	0.0001	0.0026	0.0003	0.0013	2.9177e-05

*The results express the mean value μ and standard deviation σ of the 20 tests run for the four experiments*.

### 3.1. Wrist Prosup

In the details of Figures 4A,B, the corrective action of the cerebellar-like circuit (Experiments II, IV) leads ϑ_*c*, 0_ faster to the desired trajectory ϑ_*r*, 0_ with respect to the case without corrections (Experiments I, III). ϑ_*c*, 0_(*t*) starts getting closer to the desired position in about one period *T* = 4 s after the activation of the cerebellum (Figures 4C,D). In Figures 5A,B it is evident how the angular position error *e*_ϑ_0__ drops when the cerebellum action grows (Figures 5C,D). In particular, the mean absolute error drastically decreased by the 95 and 94% in experiment II and IV respectively, while it only decreased by the 74 and 73% in Experiment I and III (Figure 10A, the numerical results are reported in Table 3). The main difference between experiments with and without ball is the σ standard deviation. In the final period, the experiments with the ball present a larger standard deviation which is 30% (without cerebellum) and 19% (with cerebellum) respect to the NO ball-case.

### 3.2. Wrist Yaw

The wrist yaw joint is the most affected by the cerebellum action. In Figure 6, it is evident how with only the PID contribution ϑ_*c*, 1_(*t*) presents a constant and large offset with respect to ϑ_*r*, 1_(*t*). As soon as the cerebellum contribution $\Delta \tau_{1}^{DCN}$ grows (around the 50 s, Figures 7C,D) the error descends (Figures 7A,B). The mean absolute error decreases by the 78% in experiment II and IV, while it only drops 1% in experiments I and III (Figure 10B). In the last period, the experiments with the ball have a standard deviation 30–33% larger than the NO ball-cases.

### 3.3. Wrist Pitch

On the other hand, the wrist pitch gains from the cerebellar action only when the error is larger than $e_{\vartheta_{2}}^{thresh}$, which is around 40–60 s (Figure 8), taking into account that the cerebellum is started at *t* = 40 s. The $\Delta \tau_{1}^{DCN}$ gets more silent (Figures 9C–E) when the angular position error is small (Figures 9A,B). In Figure 10C is more evident how the cerebellum accelerates the corrective action between iteration 10 and 15 where the MAE with the cerebellum (experiment II) is 17% lower respect to experiment I (in experiment IV the MAE is 16% lower respect to experiment III).

## 4. Discussions

In this work, a bio-mimetic control scheme is presented in the framework of a robotic task, in which simultaneous control of the object dynamics and of the internal force exerted by the robot arm to follow a trajectory with the object attached to it is required. To address multi-joint corrective responses, we induced and combined three-joint wrist motions. Thus adaptation skills are required especially to deal with an external perturbation acting on the robot-object system. The main observation is that plastic mechanisms given by a feed-forward cerebellum-like controller effectively contribute to the learning of the dynamics model of the robot arm-object system and to the adaptive corrections in terms of torque commands applied to the joints. These cerebellar torque contributions, together with feedback (PID) torque outcome, allow the progressive error reduction by incorporating distributed synaptic plasticity based on the feedback from the actual movement.

**Figure 11:**
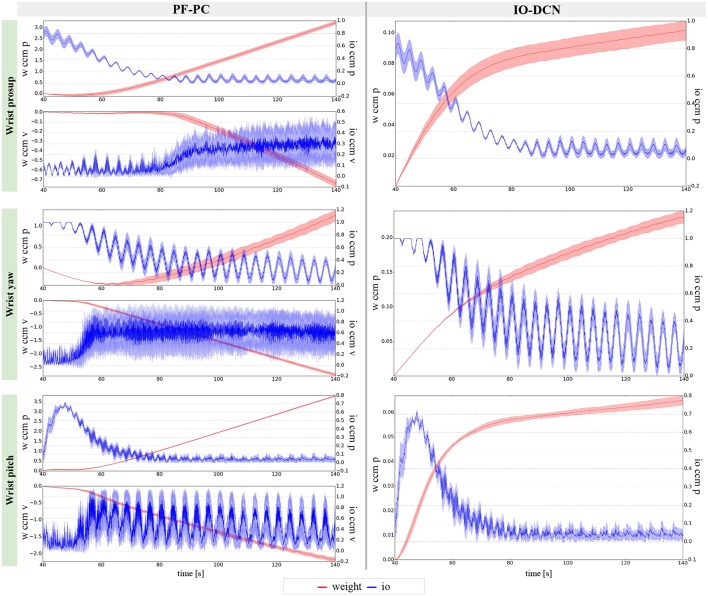
Learning evolution of the cerebellar-like network in experiment IV: influence of the inferior olive on the PC-PF parallel fibers-Purkinje cells and IO-DCN inferior olive-Deep cerebellar nuclei connections. The plots show the results of the 20 tests in terms of mean value (solid line) and 95% confidence interval (colored area).

The results about the three controlled joints showed a fast reactive control in the test cases when the cerebellum-like model is active, which is even more evident when the ball (random perturbation) is present as shown in Figures 4, 6, 8B,D. An incremental velocity control input is then provided to the controller of the system to deal with the perturbation. The purpose of considering a heterogeneous stochastic dynamical stimuli (board and ball) was to test and examine the activation of incremental learning and adaptation of the cerebellum-like controller and at the same time to confirm its coupling with the feedback control inputs. Previous studies have shown that the feedback processes are omnipresent in voluntary motor actions (Scott et al., 2015) and rapid corrective responses occur even for very small disturbances that approach the natural variability of limb motion. In human beings, these corrections commonly require increases in muscle activity generated i.e., by applied loads (Nashed et al., 2015). By analogy, a similar effect can be noticed at joint-level in our system. In the experimental situation, the joints that are more influenced by the limb dynamics (wrist prosup and yaw joints) under the effect of the table and ball increase their control input activity as represented in Figures 5, 7C,D, while the wrist pitch joint has a much more reduced activity re influenced by the limb dynamics (wrist prosup and yaw joints) under the effect of the table and ball increase their control input activity as represented in Figures 9C,D compared to the previous two joints. This phenomena is also reflected in the control input provided by the cerebellum-like model. The bigger the position error is at the beginning of the simulation with only the PID control case (experiments I and III) the more effective the cerebellar-like corrections are (experiments II and IV) as shown in Figures 5, 7, 9A,B. It should be noted that for the wrist pitch joint the PID controller leads to ~0.0 (rad) MAE around 40 s from the beginning of the simulation. However, among all the joints, the fundamental role of the cerebellum in motor control is confirmed by its anticipatory response for decreasing the error as it is appreciated in Figure 10. The control system achieved these result by creating up to 9 *Gr* receptive fields per uml at the granular level (or rather LWPR). In Figure 11, it is possible to appreciate how the IO inferior olive signals (in blue) of each ccm promptly influence the synaptic weights (in red) between the PF parallel fibers and the PC Purkinje cells (left column), and the contribution of the inferior olive itself on the DCN Deep Cerebellar nuclei corrective action (right column). In the IO-DCN connection details, the synaptic weights rapidly increment in the first tract around 40–60 s where the error is higher and then keep increasing slowly for the final adjustments. On the other hand, the PF-PC connection tends to not over-react at the beginning of the simulation around 40–60 s, while it strengthen when the error decline. We assume that this opposite influence of the IO on the synaptic weights makes possible the filtering and the dumping of any external disturbances or high error.

This control model proposes a plausible explanation on how control feedback is used by the central nervous system (CNS) to correct for intrinsic as well as external sources of disturbances. Furthermore, the bio-mimetic model represents a plausible control scheme for voluntary movements that can be generalized to control robotic agents without mayor tuning of the parameters. Our controller with distributed plasticity allows efficient adjustment of the corrective signal regardless of the dynamic features of the robot arm and of the way the added perturbations affect the dynamics of the arm plant involved. According to this, the controller (cerebellum-like and PID) is adaptable by providing adjustable torque commands among the joints to overcome external dynamic and stochastic perturbations and to have a both fast and precise movement. This replies to our question about if the sensory-motor information extrapolation made by the cerebellum-like facilitates motor prediction and adaptation in changing conditions. It should be noted that the adaptation mechanism adopted here is not constrained to any specific plant or testing framework, and could therefore be extrapolated to other common testing paradigms.

D'Angelo et al. (2016) illustrated in their paper the schematic representation of how the core cerebellar microcircuit is wired inside the whole brain. The proposed cerebellar-like model has been designed in analogy with it. In contrast with Garrido Alcazar et al. (2013), Casellato et al. (2014), Antonietti et al. (2017), the proposed model encodes the movement kinematics at the mossy fibers level (Ebner et al., 2011), and presents a coupling at the Purkinje layer for velocity and position terms representation. Likewise, the synaptic strengths at PC-DCN level as well the synaptic strengths at IO-DCN level are modulated by signals related to position or velocity. The mossy fibers are connected to the DCN and to some granular cells to convey the efference copy or motor command information. The IO cells are devoted to teaching signal error transmission in terms of position and velocity errors. The teaching errors modulate the synaptic strengths at PF-PC and IO-DCN levels.

Tokuda et al. (2017) postulated that high dimensionality problem (high-dimensional sensory-motor inputs vs. low training data) is accomplished by the cerebellum by regulating the synchronous firing activities of the inferior olive (IO) neurons. Though the implementation of coupling mechanisms at the inferior olive cells would be an interesting work to have a better explanation on multiple joint control. This extension could also provide additional insights into the internal connectivity of the cerebellar microcomplex. Further investigation will be possible in the future of how specific properties of the cells, of the network topology and synaptic adaptation mechanisms complement each other in the bio-inspired architecture.

### 4.1. Neural Basis of Feedback Control for Voluntary Movements

Feedback control of movement is essential to guarantee movement success especially to compensate for perturbation arising from the interaction with the external world. Different brain areas (primary motor cortex, primary somatosensory cortex, cerebellum, supplementary motor area, etc.) are involved during a voluntary movement and cooperate in many levels of hierarchy. Feedback control theory might be the key for understanding how the previous areas plan and control the movement hierarchically. By using control terminology, during the voluntary movement of a limb, the primary motor cortex acts as a controller, and the limb connected to neuronal circuits becomes the controlled object.

The cerebellum learns and provides the internal models that reproduce the inverse or direct dynamics of the body part. Thanks to the cerebellar internal model learning, the primary motor cortex performs the control without an external feedback (Koziol et al., 2014). By our simulations, we suggest that such behavior can be confirmed. Indeed, the cerebellar-like contributions drive the feedback controller toward better accuracy and precision of the movement. In the future, a visual feedback input will be considered to probe the sophistication of feedback control processing and cerebellar-like learning consolidation.

## Author Contributions

MC and ST conceived and designed the experiments, analyzed the data, and wrote the paper. MC implemented the architecture and performed the experiments. EA contributed to materials and analysis tools. HL and EF reviewed the paper.

### Conflict of Interest Statement

The authors declare that the research was conducted in the absence of any commercial or financial relationships that could be construed as a potential conflict of interest.

## Footnotes

^1^*w*^*pf*−*pc*^ weighting kernel parameters: *LTD*_*max*_ = 10^−3^, *LTP*_*max*_ = 10^−3^, α = 170.

^2^*w*^*pc*−*dcn*^ weighting kernel parameters: *LTD*_*max*_ = 10^−4^, *LTP*_*max*_ = 10^−4^, α = 2.

^3^*w*^*mf*−*dcn*^ weighting kernel parameters: *LTD*_*max*_ = 10^−4^, *LTP*_*max*_ = 10^−4^, α = 2.

^4^$w_{n,p}^{io-dcn}$ weighting kernel parameters: *MTD_max_* = −10^−4^, *MTP_max_* = −10^−5^, α = 100.
